# Comparative Analysis of Plasma Protein Dynamics in Women with ST-Elevation Myocardial Infarction and Takotsubo Syndrome

**DOI:** 10.3390/cells13211764

**Published:** 2024-10-24

**Authors:** Shafaat Hussain, Sandeep Jha, Evelin Berger, Linnea Molander, Valentyna Sevastianova, Zahra Sheybani, Aaron Shekka Espinosa, Ahmed Elmahdy, Amin Al-Awar, Yalda Kakaei, Mana Kalani, Ermir Zulfaj, Amirali Nejat, Abhishek Jha, Tetiana Pylova, Maryna Krasnikova, Erik Axel Andersson, Elmir Omerovic, Björn Redfors

**Affiliations:** 1 Department of Molecular and Clinical Medicine, Institute of Medicine, University of Gothenburg, 413 45 Gothenburg, Sweden; sandeep.jha@vgregion.se (S.J.); linnea.molander@gu.se (L.M.); valentyna.sevastianova@wlab.gu.se (V.S.); zahra.sheybani@gu.se (Z.S.); aaron.espinosa@gu.se (A.S.E.); ahmed.elmahdy@wlab.gu.se (A.E.); amin.al-awar@gu.se (A.A.-A.); yalda.kakaei@gu.se (Y.K.); mana.kalani@wlab.gu.se (M.K.); ermir.zulfaj@gu.se (E.Z.); amirali.nejat@wlab.gu.se (A.N.); abhishek.jha@gu.se (A.J.); tetiana.pylova@wlab.gu.se (T.P.); maryna.krasnikova@wlab.gu.se (M.K.); axel.andersson@wlab.gu.se (E.A.A.); elmir@wlab.gu.se (E.O.); bjoern.redfors@wlab.gu.se (B.R.); 2 Wallenberg Centre for Molecular and Translational Medicine, Institute of Medicine, University of Gothenburg, 405 30 Gothenburg, Sweden; 3 Department of Cardiology, Sahlgrenska University Hospital, 413 45 Gothenburg, Sweden; 4 Proteomics Core Facility, Sahlgrenska Academy, University of Gothenburg, 405 30 Gothenburg, Sweden; evelin.berger@gu.se

**Keywords:** ST-elevation myocardial infarction, Takotsubo syndrome, plasma proteomics, temporal changes, energy metabolism, tissue remodeling, biomarkers

## Abstract

Background: ST-elevation myocardial infarction (STEMI) and Takotsubo syndrome (TS) are two distinct cardiac conditions that both result in sudden loss of cardiac dysfunction and that are difficult to distinguish clinically. This study compared plasma protein changes in 24 women with STEMI and 12 women with TS in the acute phase (days 0–3 post symptom onset) and the stabilization phase (days 7, 14, and 30) to examine the molecular differences between these conditions. Methods: Plasma proteins from STEMI and TS patients were extracted during the acute and stabilization phases and analyzed via quantitative proteomics. Differential expression and functional significance were assessed. Data are accessible on ProteomeXchange, ID PXD051367. Results: During the acute phase, STEMI patients showed higher levels of myocardial inflammation and tissue damage proteins compared to TS patients, along with reduced tissue repair and anti-inflammatory proteins. In the stabilization phase, STEMI patients exhibited ongoing inflammation and disrupted lipid metabolism. Notably, ADIPOQ was consistently downregulated in STEMI patients in both phases. When comparing the acute to the stabilization phase, STEMI patients showed increased inflammatory proteins and decreased structural proteins. Conversely, TS patients showed increased proteins involved in inflammation and the regulatory response to counter excessive inflammation. Consistent protein changes between the acute and stabilization phases in both conditions, such as SAA2, CRP, SAA1, LBP, FGL1, AGT, MAN1A1, APOA4, COMP, and PCOLCE, suggest shared underlying pathophysiological mechanisms. Conclusions: This study presents protein changes in women with STEMI or TS and identifies ADIPOQ, SAA2, CRP, SAA1, LBP, FGL1, AGT, MAN1A1, APOA4, COMP, and PCOLCE as candidates for further exploration in both therapeutic and diagnostic contexts.

## 1. Introduction

ST-elevation myocardial infarction (STEMI) and Takotsubo syndrome (TS) have distinct pathophysiologies and both result in the sudden loss of myocardial function. Whereas STEMI occurs as a consequence of an acute coronary artery occlusion [1,2], TS occurs without a coronary occlusion and is believed to be caused by severe emotional or physical stress [3] Despite their distinctly different mechanisms, STEMI and TS have overlapping clinical presentations and are difficult to distinguish without an invasive coronary angiogram [4].

A better understanding of the plasma profiles in STEMI and TS is important for better differentiating between TS and STEMI and for better understanding the mechanisms behind cardiac dysfunction in these two conditions in order to develop effective diagnostic and therapeutic tools [1,2,4]. Modern proteomics is a powerful tool to elucidate the dynamic protein expression changes and molecular pathways behind these disorders [5]. Prior research on plasma samples has identified key proteins and pathways linked to myocardial infarction and heart failure, unveiling potential therapeutic targets and diagnostic markers [6]. Plasma serves as a readily accessible biomarker reservoir that mirrors the body’s physiological and pathological states [7]. Nonetheless, comparative plasma proteomic research focusing on STEMI and TS, along with their temporal progression, remains scarce. Analyzing proteomic profiles of STEMI and TS patients can offer insights into the driving processes behind their development and progression.

Therefore, the objective of this study was to compare the changes in the plasma proteome in female STEMI and TS patients during the acute phase (days 0–3 post symptom onset) and the stabilization phase (days 7, 14, and 30) to understand the molecular differences between these conditions.

## 2. Methods

### 2.1. Study Design

This study focused on women with no prior history of myocardial infarction or known pre-existing wall motion abnormalities that presented with either STEMI (*n* = 24) or TS (*n* = 12), as per the diagnostic criteria outlined by the European Society of Cardiology [8], at Sahlgrenska University Hospital in Gothenburg, Sweden, within the framework of the Stunning in Takotsubo versus Acute Myocardial Infarction study (STAMI, NCT04448639).

### 2.2. Participant Recruitment and Selection

All women included in this study were recruited within a 12 h period after symptom onset. Only patients with no prior history of myocardial infarction or known pre-existing wall motion abnormalities were considered for inclusion, as documented in their medical records and confirmed during clinical evaluation. Additionally, patients diagnosed with STEMI were required to undergo primary percutaneous coronary intervention (PCI) within 6 h of symptom onset. Informed consent was obtained from each participant, and this study adhered to the principles set forth in the Declaration of Helsinki. This study was approved by the Swedish Ethical Review Authority (registration number 2022-01003-02). All patient data were handled following the EU Data Protection Directive.

### 2.3. Baseline Characteristics

Time of symptom onset was obtained from an interview with each patient according to a predefined questionnaire. Baseline characteristics, including demographic information and medical history, were obtained from the patients and their medical charts. Results from the diagnostic work-up and clinical variables were registered consecutively as patients were enrolled in this study.

### 2.4. Blood Sampling and Plasma Collection

Trained personnel collected blood samples from the study participants at two specific time points:

Acute phase (days 0–3): Blood samples for the acute phase were taken at baseline and on days 1, 2, and 3 following the onset of symptoms.

Stabilization phase (days 7, 14, and 30): Subsequent blood samples were collected on days 7, 14, and 30 post symptom onset.

**Figure 1:**
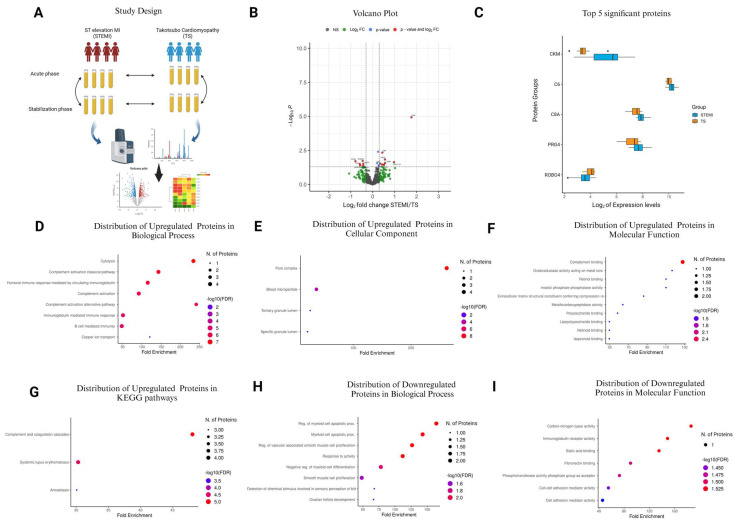
Alterations in plasma protein expression between STEMI and TS patients during the acute phase. (**A**) Study design and workflow of nLC-MS-based proteomics. (**B**) Volcano plot depicting the significant proteins in terms of their significance levels and fold changes in expression. Proteins with *p*-values less than 0.05 were considered to be statistically significant (red and blue points in the plot). Of these, those with more than a two-fold expression are additionally demarcated (proteins denoted in red). The proteins represented in black (NS) and green (log2FC) points denote the ones that were not statistically significant and were not considered for downstream analyses. (**C**) Box plots represent the top 5 most significant proteins. (**D**–**G**) Gene Ontology term enrichment analysis of upregulated proteins in STEMI compared to TS based on Biological Process, Cellular Component, Molecular Function, and KEGG Pathway. (**H**,**I**) GO term enrichment analysis of downregulated proteins in STEMI compared to TS based on Biological Process and Molecular Function. The bubble plot diagrams provide information on the top 10 pathways in terms of GO fold enrichment, significance (FDR in log10), and the number of proteins in each pathway.

The blood collection was performed using standard venipuncture techniques. Whole blood was drawn into ethylenediaminetetraacetic acid (EDTA)-coated tubes to prevent coagulation. After collection, the samples were maintained at 4 °C before centrifuging at 2000× *g* for 10 min. After the centrifugation, plasma was collected and stored at −80 °C until analysis. Proper handling and storage procedures were followed to ensure the integrity of the plasma samples for subsequent proteomic profiling analysis. The study design is shown in Figure 1A.

### 2.5. Proteomic Analysis

#### 2.5.1. Sample Preparation

Aliquots of 2 µL plasma were diluted in 50 mM triethylammonium bicarbonate (TEAB), reduced with 10 mM dithiothreitol at 60 °C for 30 min, and then alkylated using 20 mM iodoacetamide at room temperature in the dark. The reaction was quenched by additional incubation with 10 mM dithiothreitol.

For digestion, 2.6 µg LysC/trypsin (Promega, Madison, WI, USA) was added, and incubation took place overnight at 37 °C while shaking. An additional 2.6 µg trypsin (Thermo Fisher Scientific, Waltham, MA, USA) was added, and proteins were digested for another three hours. Peptide concentration was determined using the Pierce™ Quantitative Fluorometric Peptide Assay (Thermo Scientific). Peptides were diluted in 0.1% formic acid (FA) and approximately 500 ng peptides was loaded onto Evotips Pure (Evosep, Odensen, Denmark) according to the manufacturer’s instructions.

#### 2.5.2. Identification and Quantification

The liquid chromatography (LC) system Evosep One was used running the 30 samples per day (30SPD) method on a Pepsep C18 column (15 cm × 150 µm ID, 1.5 µm particle size). The timsTOF HT was run in DIA-PASEF mode with variable isolation windows, which were created using py_diAID (0.0.18) [9] with the recommended default settings based on an in-house spectral library of depleted and crude plasma.

The raw data were matched using directDIA (homo sapiens, Swissprot, June 2023, 20,407 entries) within the Spectronaut software (18.5). For identification, one missed cleavage with trypsin was accepted, oxidation of methionine and acetylation of protein *n*-terminus were set as variable, and carbamidomethylation of cysteine was set as a fixed modification. Quantification was performed on Only Protein Group Specific on the MS2 level, and automatic cross-run normalization was enabled.

#### 2.5.3. Statistical Analysis

Differential expression analysis was conducted in R version 4.3.2 using a two-sample *t*-test and paired *t*-test on log-transformed data to identify proteins with significant differences. To control multiple testing, the Benjamini–Hochberg procedure was employed. Proteins with a false discovery rate (FDR) value below 0.05 were considered differentially expressed. Principal component analysis served as quality control for sample assessment and group clustering.

### 2.6. Bioinformatic Analysis

Gene Ontology (GO) enrichment was performed on the proteins that were differentially expressed using the ShinyGo 0.76 platform from South Dakota State University [10]. This enrichment analysis was based on a fold enrichment method derived from the hypergeometric distribution, and statistical reliability was enhanced by false discovery rate (FDR) correction. The complete set of protein-coding genes in the human genome was used as a reference for the analysis. To facilitate interpretation, proteins were categorized as upregulated or downregulated, and their distinctions were displayed in lollipop charts. These charts depict the enrichment of each GO term across biological processes, cellular components, molecular functions, and KEGG pathways. The FDR threshold was set at <0.05, and only GO terms associated with a minimum of 10 differentially expressed pathways were showcased. This approach was chosen to emphasize biologically pertinent enrichments while minimizing potential data noise. Volcano plots were subsequently generated using R (version 4.2.2) with the EnhancedVolcano package.

## 3. Results

### 3.1. Patient Characteristics

The baseline clinical characteristics of STEMI (*n* = 24) and TS (*n* = 12) patients are presented in Table 1. The study cohort comprised postmenopausal women without pre-existing cardiac dysfunction matched by age. The patients were also comparable in terms of BMI. Hypertension was slightly more prevalent in TS (41.7%) compared to STEMI patients (33.3%). Among the STEMI patients, 12.5% had COPD, while none of the TS patients had a history of COPD. Smoking, which is a main risk factor for COPD, had a higher prevalence in STEMI patients (20.8%) compared to TS (8.3%). Similarly, a higher percentage of STEMI patients were former smokers (20.8%) compared to TS (8.3%). On admission, oxygen supplementation was required in a higher proportion of TS (50.0%) than in STEMI patients (18.5%).

### 3.2. Clinical Outcomes

The clinical outcomes for patients with STEMI (*n* = 24) and TS (*n* = 12) are summarized in Table 2. Pre-procedure complications were minimal, with sustained ventricular tachycardia absent in both groups. However, ventricular fibrillation was observed in 1 STEMI patient (4.2%), and grade III AV block was noted in another STEMI patient (4.2%), while both conditions had instances of sinus bradycardia (12.5% in STEMI and 8.3% in TS). Cardiogenic shock was significantly more prevalent in TS patients (25.0%) compared to STEMI patients (4.2%). Post-procedure, there were no reports of sustained ventricular tachycardia or ventricular fibrillation in either group within the first three days. There was one case of grade III AV block in a STEMI patient (4.2%) and one instance of cardiogenic shock in a STEMI patient (4.2%) alongside two cases (16.7%) in the TS group. Importantly, there were no deaths reported in either group during the first three days. At the 30-day follow-up, there were still no fatalities in either cohort, although one STEMI patient (4.2%) required rehospitalization for heart failure, while no TS patients experienced such a need. Additionally, neither group reported any subsequent myocardial infarctions, strokes, transient ischemic attacks (TIAs), or thromboembolic events during the follow-up period. Overall, these results indicate that both STEMI and TS patients had low rates of serious complications and demonstrated differences in certain pre- and post-procedure outcomes, particularly with respect to cardiogenic shock.

### 3.3. Laboratory Markers in the Acute Phase

The laboratory markers for patients with STEMI (*n* = 24) and TS (*n* = 12) are detailed in Table 3. The peak high-sensitivity cardiac troponin I (hs-cTnI) levels were significantly elevated in STEMI patients, measuring 32,000 ng/L (IQR: 25,000 to 46,000 ng/L), compared to 1700 ng/L (IQR: 550 to 2300 ng/L) in TS patients. Similarly, peak high-sensitivity cardiac troponin T (hs-cTnT) levels were markedly higher in STEMI patients at 2630 ng/L (IQR: 1985 to 4460 ng/L) versus 280 ng/L (IQR: 196 to 433.5 ng/L) in TS patients. In terms of cardiac stress markers, peak NT-proBNP levels were elevated in TS patients, measuring 6140 ng/L (IQR: 4990 to 7620 ng/L), compared to 2540 ng/L (IQR: 2020 to 4180 ng/L) in STEMI patients. Baseline creatinine levels were comparable, with STEMI patients at 73 μmol/L (IQR: 60 to 84 μmol/L) and TS patients at 68.5 μmol/L (IQR: 55.8 to 84.2 μmol/L). Baseline cholesterol levels were slightly lower in STEMI patients (5.2 mmol/L, IQR: 4.4 to 6.0) compared to TS patients (5.7 mmol/L, IQR: 4.9 to 6.0). Baseline low-density lipoprotein (LDL) levels were also similar, at 3.8 mmol/L (IQR: 3.2 to 4.6) for STEMI and 3.6 mmol/L (IQR: 3.0 to 4.5) for TS. However, high-density lipoprotein (HDL) levels were higher in TS patients (1.4 mmol/L, IQR: 1.3 to 1.8) compared to STEMI patients (1.2 mmol/L, IQR: 1.0 to 1.4), while baseline triglycerides were comparable at 0.9 mmol/L (IQR: 0.8 to 1.1) in STEMI patients and 0.8 mmol/L (IQR: 0.8 to 1.2) in TS patients.

### 3.4. Plasma Proteome of Women with STEMI and TS

A total of 735 proteins from plasma were initially identified and quantified across 36 women STEMI and TS patient samples. After filtering, we excluded proteins if any of the groups in that analysis had more than 30% missing values. Consequently, the number of proteins varied between analyses, as the data in each analysis differed. For instance, in the acute comparison between STEMI and TS, we filtered a total of 467 proteins, whereas in the stabilization comparison, the number was 476 proteins. Moreover, in the paired comparisons for both STEMI and TS, the total number of proteins varied, with 485 for paired STEMI and 460 for paired TS. The majority of these filtered proteins were annotated as plasma proteins according to Gene Ontology. However, some proteins had different annotations, such as heart, liver, kidney, brain, and other tissues. This indicates the secretion of these proteins into the bloodstream in the diseased condition, suggesting they may serve as interesting biomarkers.

### 3.5. Comparison of STEMI vs. TS in the Acute Phase

A volcano plot was used to display the expression differences between STEMI and TS in plasma samples, based on log2-fold change versus *p*-value (Figure 1B). Out of 467 quantified plasma proteins, 18 exhibited significant alterations: 12 were upregulated, and 6 were downregulated in STEMI compared to TS (Figure 1B and Table 4). Notably, proteins CKM, C6, C8A, PRG4, and ROBO4 were identified as the top five most significantly changed proteins (Figure 1C). Through GO enrichment analysis, several biological processes, cellular components, molecular functions, and KEGG pathways revealed significant changes (Figure 1D–I and Figure S1). Upregulated proteins in STEMI patients, such as C8B, C6, C8A, and C8G, were found to be prominently involved in processes like cytolysis, classical complement activation, and the humoral immune response (Figure 1D and Table S1).

These alterations were characterized by substantial fold enrichments, reaching up to 261.73 in the pore complex (Figure 1E and Table S1). Complement binding activities, particularly involving C8G and C8A, were evident in molecular function analysis (Figure 1F and Table S1). Furthermore, KEGG pathway analysis underscored the involvement of proteins like C6, C8A, C8B, and C8G in pathways such as complement and coagulation cascades (Figure 1G and Table S1). Conversely, downregulated proteins in STEMI patients, including MMP2 and ADIPOQ, exhibited reduced representation in processes related to apoptosis, smooth muscle cell proliferation (Figure 1H and Table S1), and molecular functions like carbon nitrogen lyase activity and immunoglobulin receptor activity (Figure 1I and Table S1).

### 3.6. Comparison of STEMI vs. TS in the Stabilization Phase

**Figure 2:**
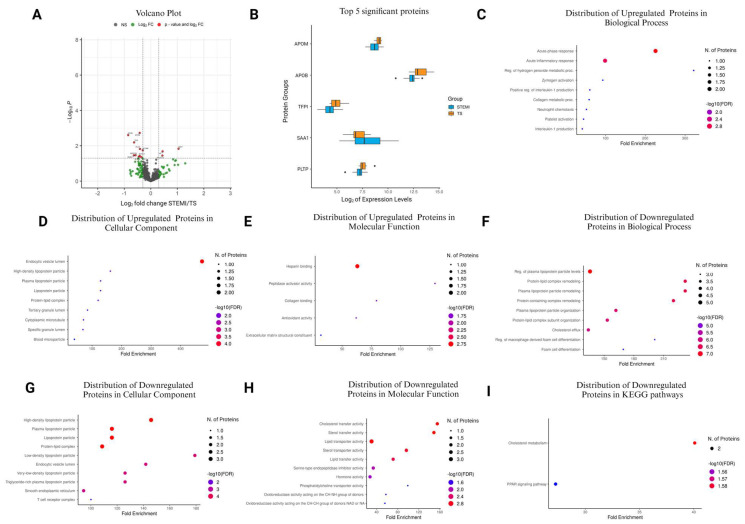
Alterations in plasma protein expression between STEMI and TS patients during the stabilization phase. (**A**) Volcano plot representing proteins based on significance and expression fold changes. Statistically significant proteins (*p* < 0.05) are highlighted in red and blue, with those exceeding two-fold change in red. Non-significant proteins are shown in black (NS), and proteins not meeting fold change criteria are in green (log2FC). (**B**) Box plots illustrating the expression levels of the top 5 most significant proteins. (**C**–**E**) GO enrichment analysis for upregulated proteins in STEMI, categorized by Biological Process, Cellular Component, and Molecular Function. (**F**–**I**) GO enrichment for downregulated proteins, encompassing Biological Process, Cellular Component, Molecular Function, and KEGG Pathway. The bubble plot diagrams highlight the top 10 enriched pathways with details on fold enrichment, significance, and protein count.

During the stabilization phase, the evaluation of plasma protein expression between STEMI and TS revealed significant alterations, illustrated by a volcano plot analysis (Figure 2A). Among the 476 quantified proteins, 13 displayed notable changes, with 3 showing upregulation and 10 showing downregulation (Figure 2A and Table 5). Proteins APOM, APOB, TFPI, SAA1, and PLTP were found to be the top five most significant changed proteins (Figure 2B).

In the GO Biological Process category, proteins like SAA1 and HP were significantly enriched in processes such as the acute-phase response and acute inflammatory response, with fold enrichments reaching up to 225.70 (Figure 2C and Table S2). Similarly, in the GO Cellular Component category, SAA1 and HP were prominent in the endocytic vesicle lumen, exhibiting a fold enrichment of 472.58 (Figure 2D and Table S2). In the GO Molecular Function category, PCOLCE and SAA1 displayed enrichment in functions like heparin binding, with a fold enrichment of 62.96 (Figure 2E and Table S2). Conversely, in the GO Biological Process category, proteins such as PLTP, NA, APOB, and AGT were downregulated, particularly in processes related to plasma lipoprotein particle levels’ regulation and protein-containing complex remodeling, with fold enrichments reaching as high as 231.80 (Figure 2F and Table S2).

Similarly, in the GO Cellular Component category, APOB, NA, and PLTP were downregulated in high-density lipoprotein particle, protein–lipid complex, and plasma lipoprotein particle components, with fold enrichments ranging from 108.59 to 145.82 (Figure 2G and Table S2). Additionally, in the GO Molecular Function category, APOB and PLTP showed downregulation in functions such as sterol and cholesterol transfer activity, with fold enrichments ranging from 148.75 to 155.22 (Figure 2H and Table S2). Finally, in the KEGG pathway analysis, the cholesterol metabolism pathway (hsa04979) demonstrated downregulation, with proteins APOB and PLTP showing a fold enrichment of 40.08 (Figure 2I and Table S2).

### 3.7. Comparison of STEMI Acute Phase vs. STEMI Stabilization Phase

**Figure 3:**
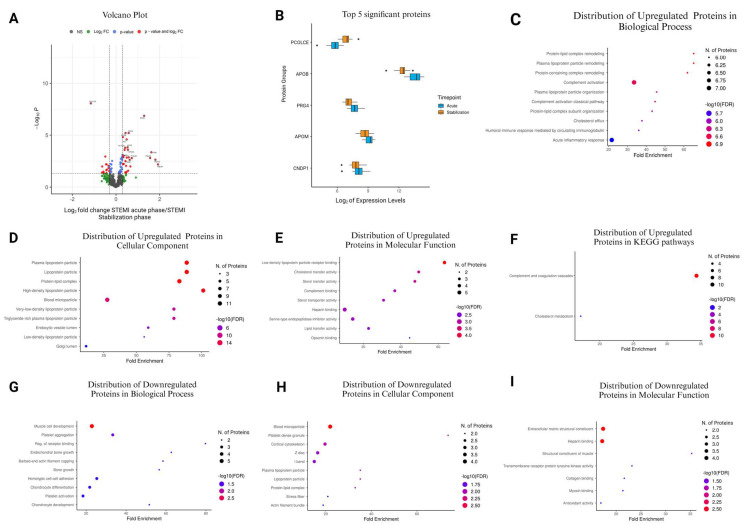
Plasma protein changes in patients with STEMI between the acute phase and stabilization. (**A**) Volcano plot displays proteins by their significance and fold changes. Proteins with *p* < 0.05 are shown in red (those with over two-fold change) and blue. Proteins not meeting significance are in black (NS), and those not reaching the fold change threshold are in green (log2FC). (**B**) Box plots of the top 5 significantly altered proteins. (**C**–**F**) GO enrichment analysis of upregulated proteins, categorized by Biological Process, Cellular Component, Molecular Function, and KEGG Pathway. (**G**–**J**) GO analysis for downregulated proteins, broken down into Biological Process, Cellular Component, Molecular Function, and KEGG Pathway. Bubble plot diagrams spotlight the top 10 enriched pathways, detailing fold enrichment, significance level, and protein constituents.

A volcano plot analysis unveiled significant changes in 74 proteins (50 upregulated, 24 downregulated) when comparing the STEMI acute phase to the stabilization phase (Figure 3A and Table 6). Notably, the top five most significant proteins, PCOLCE, APOB, PRG4, APOM, and CNDP1, showed marked alterations in expression levels (Figure 3B).

In the GO Biological Process category, proteins such as MASP2, CRP, FCN3, and MBL2 were significantly enriched in processes like complement activation, with fold enrichments reaching up to 33.50 (Figure 3C and Table S3). Similarly, in the GO Cellular Component category, proteins like APOB, CETP, APOA5, and APOA1 were highlighted in plasma lipoprotein particles, exhibiting a fold enrichment of 88.60 (Figure 3D and Table S3). Additionally, in the GO Molecular Function category, proteins such as APOB, APOE, APOA5, and CRP displayed enrichment in functions like lipoprotein particle receptor binding, with a fold enrichment of 63.30 (Figure 3E and Table S3). In the KEGG pathways, the complement and coagulation cascades showed upregulation with proteins such as MASP2, F5, F7, F9, MBL2, and others (Figure 3F and Table S3).

Conversely, in the GO Biological Process category, proteins like ACTN1, MYL9, CSRP1, COMP, and PI16 were downregulated, particularly in processes related to muscle cell development, with a fold enrichment of 22.84 (Figure 3G and Table S3). Similarly, in the GO Cellular Component category, proteins such as APOA4, GC, GSN, and CFHR1 were downregulated in blood microparticles, with a fold enrichment 21.93 (Figure 3H and Table S3). Additionally, in the GO Molecular Function category, proteins like COMP, PCOLCE, EFEMP1, and ECM1 exhibited downregulation in functions such as extracellular matrix structural constituents, with a fold enrichment of 17.35 (Figure 3I and Table S3).

### 3.8. Comparison of TS Acute Phase vs. TS Stabilization Phase

**Figure 4:**
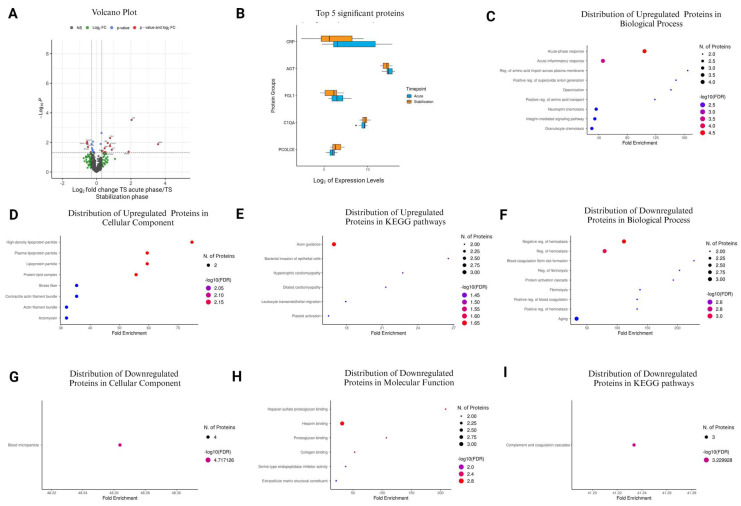
Plasma protein changes in patients with TS between the acute phase and stabilization. (**A**) Volcano plot representing proteins based on significance and expression fold changes. Statistically significant proteins (*p* < 0.05) are highlighted in red and blue, with those exceeding two-fold change in red. Non-significant proteins are shown in black (NS), and proteins not meeting fold change criteria are in green (log2FC). (**B**) Box plots showing the top 5 significant protein changes. (**C**–**E**) Gene Ontology term enrichment analysis of upregulated proteins in the TS acute phase compared to the TS stabilization phase based on Biological Process, Cellular Component, and KEGG Pathway. (**F**–**I**) GO term enrichment analysis of downregulated proteins in TS acute phase compared to TS stabilization phase based on Biological Process, Cellular Component, Molecular Function, and KEGG Pathway. The lollipop diagrams provide information on the top 10 pathways in terms of GO fold enrichment, significance (FDR in log10), and the number of proteins in each pathway.

A volcano plot analysis unveiled significant changes in protein expression between the acute phase and stabilization phase for TS patients (Figure 4A). Out of the 460 quantified proteins, 25 exhibited notable alterations, with 13 being upregulated and 12 downregulated (Figure 4A and Table 7). The top five upregulated proteins were CRP, AGT, FGL1, C1QA, and PCOLCE (Figure 4B). In the GO Biological Process category, upregulated proteins such as LBP, CRP, SAA2, and SAA1 showed significant enrichment in processes related to the acute-phase response and acute inflammatory response, with fold enrichments ranging up to 104.17 (Figure 4C and Table S4). In the GO Cellular Component category, upregulated proteins such as SAA2 and SAA1 were prominently associated with protein–lipid complexes, plasma lipoprotein particles, and high-density lipoprotein particles, with fold enrichments ranging from 55.69 to 74.78 (Figure 4D and Table S4).

Moreover, ILK and MYL12A were enriched in stress fibers and actin filament bundles, with fold enrichments of 35.37 and 31.92, respectively (Figure 4D and Table S4). In the KEGG pathways, upregulated proteins like MYL12A, ILK, and ITGB1 were involved in pathways such as axon guidance, with a fold enrichment 16.94 (Figure 4E and Table S4). On the contrary, in the GO Biological Process category, downregulated proteins such as HRG, F12, and COMP showed enrichment in processes related to the negative regulation of hemostasis and regulation of fibrinolysis, with fold enrichments reaching up to 111.33 (Figure 4F and Table S4). In the GO Cellular Component category, downregulated proteins like TFRC, APOA4, and HRG were associated with blood microparticles, exhibiting a fold enrichment of 48.26 (Figure 4G and Table S4). In the GO Molecular Function category, downregulated proteins such as COMP, HRG, and PCOLCE were involved in functions like heparan sulfate proteoglycan binding and heparin binding, with fold enrichments ranging from 31.48 to 208.78 (Figure 4H and Table S4). Furthermore, in the KEGG pathways, downregulated proteins such as F12, F13B, and C1QA were associated with complement and coagulation cascades, with a fold enrichment of 41.23 (Figure 4I and Table S4).

### 3.9. Consistent Proteomic Changes in the Acute Phase and Stabilization Phase, and Comparisons of STEMI and TS

**Figure 5:**
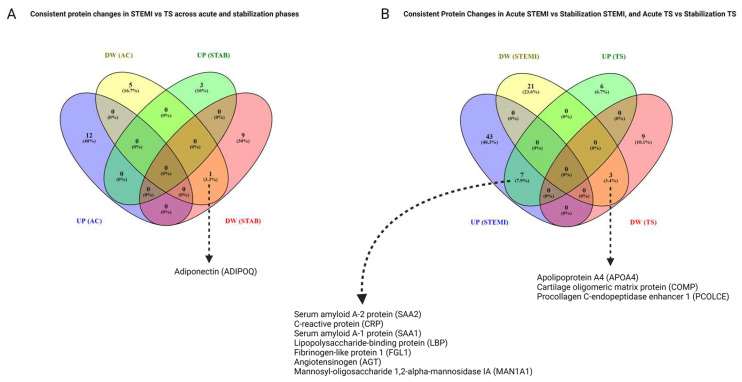
Venn diagrams showing the overlap of differentially expressed plasma proteins quantified in the study comparisons. (**A**) Overlapping upregulated (UP) and downregulated (DW) proteins between patients with STEMI and patients with TS in the acute phase (AC) and stabilization phase (STAB). (**B**) Overlapping UP and DW proteins between patients with STEMI AC and STAB and between patients with TS AC and STAB.

In comparing proteomic responses between the acute and stabilization phases in STEMI and TS patients, one protein, adiponectin (ADIPOQ), consistently showed downregulation in STEMI patients compared to TS at both time points (Figure 5A).

### 3.10. Common Protein Changes in STEMI Acute Phase vs. Stabilization Phase and TS Acute Phase vs. Stabilization Phase

In this analysis, we found seven proteins—serum amyloid A-2 protein (SAA2), C-reactive protein (CRP), serum amyloid A-1 protein (SAA1), lipopolysaccharide-binding protein (LBP), fibrinogen-like protein 1 (FGL1), angiotensinogen (AGT), and mannosyl-oligosaccharide 1,2-alpha-mannosidase IA (MAN1A1)—that exhibited consistent upregulation when comparing the acute phase to the stabilization phase in both STEMI and TS patients. In contrast, three proteins—apolipoprotein A4 (APOA4), cartilage oligomeric matrix protein (COMP), and procollagen C-endopeptidase enhancer 1 (PCOLCE)—displayed consistent downregulation across both STEMI and TS patients when comparing the acute phase to the stabilization phase (Figure 5B).

## 4. Discussion

This study investigated the dynamic proteomic differences in the blood plasma between STEMI and TS patients during both the acute and stabilization phases. We report several observations, some of which are reflective of well-known differences between the two conditions and some of which have not previously been reported. (1) STEMI patients showed increased inflammation and tissue damage proteins, coupled with deregulated tissue repair and anti-inflammatory proteins in acute-phase versus TS patients. (2) During stabilization, ongoing inflammation and disrupted lipid metabolism were evident in STEMI patients compared to TS patients. (3) Acute phase analysis of the stabilization phase revealed increased inflammatory proteins and plasma lipoprotein particles, alongside decreased muscular and structural integrity proteins and extracellular matrix proteins in STEMI patients. (4) TS patients in the acute phase compared to the stabilization phase exhibited increased proteins involved in inflammation, stress fibers, and actin filament bundles, with a regulatory response to counter excessive inflammation. (5) ADIPOQ consistently showed downregulation in STEMI patients compared to TS patients at both time points. (6) Several proteins demonstrated consistent changes across both STEMI and TS patients during the acute to stabilization phase transition, pointing to shared pathophysiological mechanisms.

In the acute phase, the upregulation of proteins such as CKM, C6, and C8A in STEMI patients compared to TS patients reflects the activation of pathways involved in tissue damage, inflammation, and the immune response [11,12,13]. CKM, a cytosolic enzyme primarily found in cardiac muscle, is released into the bloodstream following myocardial injury, serving as a biomarker for myocardial infarction [11]. The complement system, represented by proteins C6 and C8A, plays a crucial role in inflammation and the immune response by facilitating the clearance of damaged cells and pathogens [12,13]. The dysregulation of these pathways in STEMI patients underscores the magnitude of myocardial injury and the inflammatory cascade triggered by acute ischemia–reperfusion injury [11,12,13].

Conversely, the downregulation of proteins like MMP2 and ADIPOQ in STEMI patients compared to TS patients in the acute phase implicates disturbances in extracellular matrix remodeling and adipokine signaling pathways [14,15]. MMP2, a matrix metalloproteinase involved in tissue remodeling, is downregulated in response to acute myocardial infarction, reflecting impaired tissue repair mechanisms [14]. ADIPOQ, an adipokine with anti-inflammatory properties, exhibits reduced expression in STEMI patients, suggesting compromised cardioprotective effects mediated by adiponectin signaling [15]. The imbalance in these pathways could potentially lead to negative alterations in left ventricular structure and function, subsequently increasing the risk of heart failure in STEMI patients compared to TS patients.

During the stabilization phase, the persistent alterations in protein expression observed in STEMI patients compared TS patients are indicative of ongoing inflammatory processes and dysregulated lipid metabolism. The upregulation of acute-phase response proteins such as SAA1 and HP suggests sustained inflammation and tissue repair in response to myocardial injury in STEMI patients [16,17]. Experimental evidence suggests the cardioprotective role of APOB in enhancing survival and cardiac function post-MI [18], while numerous basic research studies have implicated PLTP in the development of atherosclerosis, with clinical studies broadly confirming its pro-atherogenic role as well [19]. It must be taken in account that the changes found in the stabilization phase are directly related to the drug therapy that the patients receive. Lipid-lowering therapy statins was administrated to all patients with STEMI, while only 50% of TS patients received such therapy (Table 1). The downregulation of APOB and PLTP in patients taking statins is a direct result of their pharmacodynamics as statins have pleiotropic effects, including anti-inflammatory effects. However, their effect on inflammation cannot completely neutralize the acute and strongly expressed inflammatory response characteristic of myocardial infarction. That is why SAA1 and HP remain upregulated in STEMI patients compared to TS patients in the stabilization phase.

During the acute phase of STEMI compared to the stabilization phase, there were notable changes in protein expression profiles, particularly in inflammatory, immune, and metabolic processes [20]. Proteins involved in the complement and coagulation cascades, like MASP2, CRP, FCN3, and MBL2, were elevated, underlining the known importance of innate immunity and inflammation early in myocardial infarction [21,22,23,24]. The activation of the complement system may serve dual roles, potentially exacerbating tissue damage while also contributing to the clearance of necrotic debris and facilitating subsequent healing processes [25]. The substantial enrichment in plasma lipoprotein particles, as evidenced by the involvement of APOB, CETP, APOA5, and APOA1, points to significant alterations in lipid metabolism during the acute phase of STEMI [26]. In contrast, the downregulation of proteins such as ACTN1, MYL9, CSRP1, COMP, and PI16, which are closely associated with muscle cell development and contractility, suggests a disruption in muscular and structural integrity within the heart in the acute phase post-infarction [27,28,29,30,31]. The decrease in proteins related to extracellular matrix structural constituents, such as COMP, PCOLCE, EFEMP1, and ECM1, further supports the notion of structural remodeling occurring in the myocardium [32,33,34,35].

In TS, the acute phase compared to the stabilization phase is marked by increased inflammatory mediators and stress hormones, leading to transient left ventricular dysfunction [36]. Upregulated acute-phase proteins like CRP, SAA2, and SAA1 enhance systemic inflammation and immune activation in response to stressors [37,38]. CRP, a sensitive marker of inflammation, reflects myocardial dysfunction severity [38]. Similarly, SAA2 and SAA1 contribute to immune responses and tissue repair [37]. In contrast, the decreased proteins in complement and coagulation cascade pathways, such as F12, F13B, and C1QA, indicate a regulatory response to mitigate excessive inflammation and coagulation. Downregulation of F12 and F13B prevents thrombus formation [39,40], while reduced C1QA dampens complement activation [41], potentially limiting immune-mediated tissue injury and myocardial damage in TS.

During the acute-phase and stabilization-phase comparisons of STEMI and TS patients, distinct proteomic patterns emerge, shedding light on shared and divergent molecular mechanisms underlying these cardiac conditions. One striking observation is the consistent downregulation of ADIPOQ in STEMI patients compared to TS patients at both time points. ADIPOQ, an adipokine with anti-inflammatory and cardioprotective properties, plays a crucial role in regulating glucose and lipid metabolism, insulin sensitivity, and endothelial function [42]. The persistent downregulation of ADIPOQ in STEMI patients suggests impaired adipose tissue function and dysregulated adipokine secretion, contributing to systemic inflammation, insulin resistance, and endothelial dysfunction observed in acute myocardial infarction [42]. Furthermore, the consistent downregulation of ADIPOQ may potentially serve as a diagnostic marker to differentiate between STEMI and TS. There is a significant demand for reliable diagnostic markers that can accurately distinguish between these conditions. However, while this observation is important, further studies are necessary to validate its efficacy in clinical practice.

In contrast, several proteins exhibit consistent upregulation or downregulation across both STEMI and TS patients when comparing the acute phase to the stabilization phase, highlighting shared pathophysiological pathways and potential therapeutic targets. Among the upregulated proteins, SAA2, CRP, SAA1, LBP, FGL1, AGT, and MAN1A1 are implicated in the acute-phase response, innate immune activation, and inflammatory signaling pathways [37,43,44,45,46,47]. SAA2 and SAA1 are acute-phase proteins produced in response to cytokine stimulation, promoting inflammation and tissue repair processes [37]. CRP serves as a sensitive biomarker of systemic inflammation and cardiovascular risk [43], while LBP enhances the recognition and clearance of bacterial endotoxins [44]. Recent studies have shown the association between circulating LBP levels and conditions such as diabetes, obesity, and cardiovascular phenotypes [44]. FGL1, AGT, and MAN1A1 contribute to immune modulation, metabolism, and glycoprotein processing, respectively [45,46,47], reflecting the dynamic interplay between immune, protein glycosylation, and metabolic pathways during the acute phase of both STEMI and TS.

Conversely, APOA4, COMP, and PCOLCE consistently exhibit downregulation across both STEMI and TS patients during the acute phase to stabilization phase transition. APOA4, a component of HDL, plays a critical role in lipid metabolism and reverse cholesterol transport and offers protection against atherosclerosis [48]. COMP is involved in extracellular matrix regulation and tissue remodeling [49], while PCOLCE modulates collagen biosynthesis and turnover [50]. The downregulation of these proteins may reflect disrupted lipid homeostasis, impaired extracellular matrix integrity, and altered collagen remodeling processes in both acute myocardial infarction and stress-induced cardiomyopathy.

The prevalence of smoking among patients with TS in our study was notably low at 8.3%, which contrasts with the approximately 17% reported in the GEIST registry (Núñez-Gil et al., 2023 [51]). This discrepancy may be attributed to the demographic composition of our cohort, which primarily consisted of postmenopausal women, differing from the more diverse population in the GEIST study. While smoking is often associated with increased inflammatory markers and adverse cardiovascular outcomes, the GEIST study noted that smokers did not experience significantly worse long-term mortality, despite longer hospital stays. This suggests that while smoking may influence the acute clinical presentation of TS, it may not directly impact long-term outcomes. Given the lower prevalence of smoking in our cohort, it is plausible that other factors, such as emotional stressors and underlying comorbidities, could be more critical in the pathophysiology of TS. Future research should explore the complex relationship between smoking, inflammatory pathways, and cardiovascular health, particularly in diverse patient populations affected by TS.

It is important to acknowledge that differences in protein expression patterns could potentially be influenced by confounding factors, including variations in individual risk profiles and the limited sample size. To mitigate these influences, we employed stringent inclusion criteria, selecting a well-matched cohort of postmenopausal women without prior myocardial infarction or known wall motion abnormalities, and ensured consistent sampling at well-defined phases of the disease. This careful approach was intended to minimize heterogeneity and maintain the robustness of our findings. Nevertheless, validating these proteomic observations in larger, multi-center cohorts would further substantiate the generalizability and clinical relevance of these results

In addition to traditional laboratory inflammatory markers, it is crucial to consider the insights provided by non-invasive imaging techniques, such as cardiac magnetic resonance, which have been shown to effectively assess inflammation in the remote myocardium. Recent studies indicate that incorporating these imaging biomarkers can enhance the prognostic stratification of STEMI patients, offering a more comprehensive perspective on myocardial inflammation and its impact on long-term outcomes [52].

Given the limitations inherent in our study, particularly the sample size and the demographic focus on postmenopausal women, it is essential to approach our conclusions with appropriate caution. Future studies should aim to validate our findings in larger, more heterogeneous populations, ensuring a robust understanding of the implications of inflammation in the context of STEMI.

### Limitations

This study was restricted to a small sample size, which may limit the generalizability of our findings to a broader population. We selected the stabilization phase time points of 7, 14, and 30 days to capture key recovery stages, reflecting significant physiological changes, such as the transition from acute inflammation to tissue repair. These time points provided insights into how patients with STEMI and TS differ during recovery. For the acute phase, we chose a 0–3-day window to capture the rapid physiological responses immediately following symptom onset. While a more detailed day-by-day analysis would have been ideal, practical constraints in sample collection and the need for robust statistical analysis led us to use broader timeframes. Although the limited number of samples for individual days prevented a thorough daily analysis, we believe that grouping the data into acute (0–3 days) and stabilization (7, 14, and 30 days) phases still yielded meaningful insights into the differences between STEMI and TS, enhancing our understanding of these conditions during the acute and recovery phases.

Additionally, while the plasma proteomic approach offers a comprehensive snapshot of protein expression, it requires accompanying functional studies to ascertain the exact roles and impacts of these proteins within the cellular and tissue environments. Future studies should aim to address these limitations, potentially incorporating larger, more diverse cohorts and integrating functional assays to validate and expand upon our findings.

## 5. Conclusions

This exploratory study provides an overview of the plasma proteomic differences between STEMI and TS throughout the acute and stabilization phases. Notably, our findings underline several key observations, including the anticipated elevation of inflammation and tissue damage proteins in STEMI patients compared to TS patients, alongside dysregulated tissue repair and anti-inflammatory proteins. Remarkably, ADIPOQ consistently showed downregulation in STEMI patients across both the acute and stabilization phases compared to TS, suggesting its potential as a diagnostic marker and underscoring its involvement in systemic inflammation and endothelial dysfunction.

Furthermore, our study identified ten consistent deregulated proteins across both conditions during the acute to stabilization phase comparison. These proteins—SAA2, CRP, SAA1, LBP, FGL1, AGT, MAN1A1, APOA4, COMP, and PCOLCE—are implicated in the acute-phase response, innate immune activation, inflammatory signaling pathways, lipid metabolism, and extracellular matrix regulation. Their consistent upregulation or downregulation suggests shared pathophysiological mechanisms between STEMI and TS, presenting novel therapeutic target candidates. These proteins may be interesting candidates for further exploration in both therapeutic and diagnostic contexts. However, we must consider the limitations of our study, particularly the small sample size and the narrow demographic focus on postmenopausal women, which may restrict the generalizability of our findings.

Future research is essential to validate these observations in larger, more diverse populations and to investigate the functional implications of the identified proteins within the cellular and tissue environments. Integrating non-invasive imaging techniques and functional assays could further enhance our understanding of the inflammatory processes involved in STEMI and TS, ultimately contributing to improved diagnostic and therapeutic strategies for these cardiac conditions.

## Figures and Tables

**Table 1:** Baseline characteristics.

Baseline Characteristics	STEMI (*n* = 24)	Takotsubo (*n* = 12)
Age (years)	68.5 ± 11.3	67.5 ± 9.7
BMI (kg/m^2^)	26.9 ± 5.3	26.4 ± 6.4
Atrial Fibrillation	0/24 (0.0%)	1/12 (8.3%)
COPD	3/24 (12.5%)	0/12 (0.0%)
Diabetes	1/24 (4.2%)	0/12 (0.0%)
Hyperlipidemia	1/24 (4.2%)	1/12 (8.3%)
Hypertension	9/24 (37.5%)	5/12 (41.7%)
Peripheral Vascular Disease	1/24 (4.2%)	0/12 (0.0%)
Former Smoker	5/24 (20.8%)	1/12 (8.3%)
Current Smoker	5/24 (20.8%)	1/12 (8.3%)
Trigger		
Somatic	N/A	4/12 (33.3%)
Emotional	N/A	4/12 (33.3%)
None	N/A	4/12 (33.3%)
Heart Rate (BPM)	78.1 ± 19.7	86.4 ± 16.7
Systolic Blood Pressure (mmHg)	130 ± 22.4	142 ± 34.9
Diastolic Blood Pressure (mmHg)	78.5 ± 13.3	86.6 ± 16.6
Oxygen at Admission	5/24 (20.8%)	6/12 (50.0%)
Baseline Drugs		
ARB	4/24 (16.7%)	6/12 (50.0%)
Beta Blocker	4/24 (16.7%)	4/12 (33.3%)
Calcium Channel Blocker	2/24 (8.3%)	1/12 (8.3%)
Corticosteroids	2/24 (8.3%)	0/12 (0.0%)
Paracetamol	2/24 (8.3%)	1/12 (8.3%)
Loop Diuretic	0/24 (0.0%)	1/12 (8.3%)
Metformin	1/24 (4.2%)	0/12 (0.0%)
ASA	0/24 (0.0%)	3/12 (25.0%)
P2Y12	0/24 (0.0%)	0/12 (0.0%)
OAC	1/24 (4.2%)	2/12 (16.7%)
Statins	2/24 (8.3%)	4/12 (33.3%)
Ezetrol	0/24 (0.0%)	1/12 (8.3%)
Discharge Drugs Taken Over 30 Days		
ASA	18/24 (75.0%)	5/12 (41.7%)
P2Y12	24/24 (100.0%)	0/12 (0.0%)
OAC	4/24 (16.7%)	4/12 (33.3%)
Statins	24/24 (100.0%)	6/12 (50.0%)

STEMI = ST-elevation myocardial infarction; SMD = standardized mean difference; BMI = body mass index; COPD = chronic obstructive pulmonary disease; ACEi = angiotensin-converting enzyme inhibitor; ARB = angiotensin receptor blocker; ASA = acetylsalicylic acid, OAC = oral anticoagulant; P2Y12i = P2Y12 receptor inhibitor.

**Table 2:** Clinical outcomes.

Clinical Outcomes	STEMI	Takotsubo
	(*n* = 24)	(*n* = 12)
Outcomes (3 days)		
Pre- or intra-procedure sustained ventricular tachycardia	0/24 (0.0%)	0/12 (0.0%)
Pre- or intra-procedure ventricular fibrillation	1/24 (4.2%)	0/12 (0.0%)
Pre- or intra-procedure AV-block grade III	1/24 (4.2%)	0/12 (0.0%)
Pre- or intra-procedure sinus bradycardia	3/24 (12.5%)	1/12 (8.3%)
Pre- or intra-procedure cardiogenic shock	1/24 (4.2%)	3/12 (25.0%)
Post-procedure sustained ventricular tachycardia within 3 days	0/24 (0.0%)	0/12 (0.0%)
Post-procedure ventricular fibrillation within 3 days	0/24 (0.0%)	0/12 (0.0%)
Post-procedure III degree AV-block within 3 days	1/24 (4.2%)	0/12 (0.0%)
Post-procedure II degree AV-block within 3 days	0/24 (0.0%)	0/12 (0.0%)
Post-procedure cardiogenic shock within 3 days	1/24 (4.2%)	2/12 (16.7%)
Post-procedure death within 3 days	0/24 (0.0%)	0/12 (0.0%)
Outcomes (30 days)		
Death within 30 days	0/24 (0.0%)	0/12 (0.0%)
HF rehospitalization within 30 days	1/24 (4.2%)	0/12 (0.0%)
Additional myocardial infarction within 30 days	0/24 (0.0%)	0/12 (0.0%)
Stroke or TIA within 30 days	0/24 (0.0%)	0/12 (0.0%)
Thromboembolization within 30 days	0/24 (0.0%)	0/12 (0.0%)

Clinical outcomes 3 and 30 days after hospital admission. Data are shown as *n* (%). HF = heart failure; TIA = transient ischemic attack.

**Table 3:** Laboratory markers in the acute phase.

Laboratory Markers	STEMI	Takotsubo
	*n* = 24	*n* = 12
Peak hs-cTnI (ng/L)	32,000 (25,000, 46,000)	1700 (550, 2300)
Peak hs-cTnT (ng/L)	2630 (1985, 4460)	280 (196, 433.5)
Peak NT-proBNP (ng/L)	2540 (2020, 4180)	6140 (4990, 7620)
Peak creatinine (μmol/L)	73 (60, 84)	68.5 (55.8, 84.2)
Baseline cholesterol (mmol/L)	5.2 (4.4, 6)	5.7 (4.9, 6)
Baseline LDL (mmol/L)	3.8 (3.2, 4.6)	3.6 (3, 4.5)
Baseline HDL (mmol/L)	1.2 (1, 1.4)	1.4 (1.3, 1.8)
Baseline triglycerides (mmol/L)	0.9 (0.8, 1.1)	0.8 (0.8, 1.2)

Laboratory markers in the acute phase; data are presented at admission (lipid-status), day 1 (peak TnI, TnT, and NT-proBNP), or the highest value during hospitalization (creatinine). Data are shown as median (Q1, Q3). hs-cTnI = high-sensitivity cardiac troponin-I; hs-cTnT = high-sensitivity cardiac troponin-T; NT-proBNP = *n*-terminal pro b-type natriuretic peptide; LDL = low-density lipoproteins; HDL = high-density lipoproteins.

**Table 4:** Comparative analysis of plasma protein expression levels in patients with STEMI vs. TS patients in the acute phase of hospitalization. Each entry provides the unique accession number; protein name; protein symbol; fold change in expression, represented as Log2FC; and statistical significance of the observed change, given as *T*-test *p*-value. Proteins with a positive Log2FC value are upregulated in STEMI compared to TS, while those with a negative Log2FC value are downregulated.

Accession No.	Protein Name	Protein Symbol	Log2FC	*p*-Value
P06732	Creatine kinase M-type	CKM	1.765922	1.18 × 10^−5^
A0A0C4DH31	Immunoglobulin heavy variable 1–18	IGHV1-18	0.969459	0.0234
Q92954	Proteoglycan 4	PRG4	0.563158	0.0148
P49913	Cathelicidin antimicrobial peptide	CAMP	0.554811	0.0277
P0DOX2	Immunoglobulin alpha-2 heavy chain	IGA2	0.513252	0.0367
P07357	Complement component C8 alpha chain	C8A	0.441414	0.00467
P07360	Complement component C8 gamma chain	C8G	0.432728	0.0345
P07358	Complement component C8 beta chain	C8B	0.346922	0.0301
P15169	Carboxypeptidase N catalytic chain	CPN1	0.272563	0.0406
Q9UNW1	Multiple inositol polyphosphate phosphatase 1	MINPP1	0.272294	0.0423
P13671	Complement component C6	C6	0.248847	0.004
P00450	Ceruloplasmin	CP	0.204697	0.0277
Q9HDC9	Adipocyte plasma membrane-associated protein	APMAP	−0.42631	0.0418
P08253	Matrix metalloproteinase-2	MMP2	−0.42791	0.031
Q8WZ75	Roundabout homolog 4	ROBO4	−0.45055	0.0189
Q15848	Adiponectin	ADIPOQ	−0.56441	0.0399
P01833	Polymeric immunoglobulin receptor	PIGR	−0.58109	0.0322
P27987	Inositol-trisphosphate 3-kinase B	ITPKB	−0.77973	0.0197

**Table 5:** Comparative analysis of plasma protein expression levels in STEMI vs. TS patients in stabilization phase of hospitalization. Each entry provides the unique accession number; protein name; protein symbol; fold change in expression, represented as Log2FC; and statistical significance of the observed change, given as *T*-test *p*-value. Proteins with a positive Log2FC value are upregulated in STEMI compared to TS, while those with a negative Log2FC value are downregulated.

Accession No.	Protein Name	Protein Symbol	Log2FC	*p*-Value
P0DJI8	Serum amyloid A-1 protein	SAA1	1.045935	0.0147
P00738	Haptoglobin	HP	0.446092	0.0206
Q15113	Procollagen C-endopeptidase enhancer 1	PCOLCE	0.438112	0.0355
Q13740	Activated leukocyte cell adhesion molecule	ALCAM	−0.30117	0.0182
P01019	Angiotensinogen	AGT	−0.35355	0.0436
P55058	Phospholipid transfer protein	PLTP	−0.40843	0.0152
Q9Y4L1	Hypoxia up-regulated protein 1	HYOU1	−0.41781	0.0371
O95445	Apolipoprotein M	APOM	−0.41958	0.00186
P30043	Flavin reductase	BLVRB	−0.45002	0.0421
Q15848	Adiponectin	ADIPOQ	−0.57104	0.0343
P10646	Tissue factor pathway inhibitor	TFPI	−0.6277	0.00644
P04278	Sex hormone-binding globulin	SHBG	−0.63928	0.0348
P04114	Apolipoprotein B-100	APOB	−0.85254	0.0025

**Table 6:** Changes in plasma protein expression during acute phase compared to stabilization phase in STEMI patients. The table presents a comprehensive list of proteins characterized by their accession number, protein name, and symbol. The variation in expression levels is quantified using the Log2FC (Log2 Fold Change), with positive values indicating upregulation in the acute phase compared to the stabilization phase, and negative values indicating downregulation. The statistical significance of these changes is represented by the *T*-test *p*-values.

Accession No.	Protein Name	Protein Symbol	Log2FC	*p*-Value
P0DJI9	Serum amyloid A-2 protein	SAA2	1.925515	0.00653
P02741	C-reactive protein	CRP	1.804223	0.00229
P06732	Creatine kinase M-type	CKM	1.619238	0.000446
P0DJI8	Serum amyloid A-1 protein	SAA1	1.567947	0.00157
P04114	Apolipoprotein B-100	APOB	1.293007	1.37 × 10⁻^7^
P18428	Lipopolysaccharide-binding protein	LBP	0.7243	0.0016
Q08830	Fibrinogen-like protein 1	FGL1	0.661358	0.0102
P10646	Tissue factor pathway inhibitor	TFPI	0.631156	0.00238
P11226	Mannose-binding protein C	MBL2	0.584484	0.00137
Q92954	Proteoglycan 4	PRG4	0.579881	6.16 × 10⁻^6^
Q5XPI4	E3 ubiquitin-protein ligase RNF123	RNF123	0.561965	0.0263
Q6Q788	Apolipoprotein A-V	APOA5	0.550032	0.000257
P02649	Apolipoprotein E	APOE	0.528982	0.000149
P11597	Cholesteryl ester transfer protein	CETP	0.493641	0.00147
P07195	L-lactate dehydrogenase B chain	LDHB	0.489102	0.0337
P01019	Angiotensinogen	AGT	0.489055	2.59 × 10⁻^5^
Q9Y4L1	Hypoxia up-regulated protein 1	HYOU1	0.459645	0.00906
P00915	Carbonic anhydrase 1	CA1	0.458761	0.0408
O95445	Apolipoprotein M	APOM	0.439817	6.28 × 10⁻^6^
O95497	Pantetheinase	VNN1	0.433483	0.00309
A0A0B4J1U3	Immunoglobulin lambda variable 1–36	IGLV1-36	0.428394	0.0382
Q9UHG3	Prenylcysteine oxidase 1	PCYOX1	0.417686	0.000164
Q9UK55	Protein Z-dependent protease inhibitor	SERPINA10	0.392472	0.000264
Q15166	Serum paraoxonase/lactonase 3	PON3	0.357544	0.0058
P33908	Mannosyl-oligosaccharide 1,2-alpha-mannosidase IA	MAN1A1	0.350637	0.000921
O14791	Apolipoprotein L1	APOL1	0.340909	0.00133
P22352	Glutathione peroxidase 3	GPX3	0.324418	0.0062
Q96KN2	Beta-Ala-His dipeptidase	CNDP1	0.320214	1.52 × 10⁻^5^
Q15582	Transforming growth factor-beta-induced protein ig-h3	TGFBI	0.296124	0.000792
P22792	Carboxypeptidase N subunit 2	CPN2	0.290145	0.00251
O75636	Ficolin-3	FCN3	0.280598	0.0015
Q8WWA0	Intelectin-1	ITLN1	0.276047	0.0189
P27169	Serum paraoxonase/arylesterase 1	PON1	0.272015	0.0013
P04070	Vitamin K-dependent protein C	PROC	0.244853	0.0125
P35858	Insulin-like growth factor-binding protein complex acid labile subunit	IGFALS	0.241407	0.00452
P15169	Carboxypeptidase N catalytic chain	CPN1	0.228361	0.0339
P08709	Coagulation factor VII	F7	0.226092	0.0313
O75882	Attractin	ATRN	0.219264	0.00193
P22891	Vitamin K-dependent protein Z	PROZ	0.210335	0.0108
O00187	Mannan-binding lectin serine protease 2	MASP2	0.205538	0.0228
P00736	Complement C1r subcomponent	C1R	0.201275	0.0041
P00740	Coagulation factor IX	F9	0.199953	0.00631
P12259	Coagulation factor V	F5	0.189057	0.0417
P07360	Complement component C8 gamma chain	C8G	0.181396	0.035
P07358	Complement component C8 beta chain	C8B	0.180535	0.029
P06276	Cholinesterase	BCHE	0.17415	0.0479
P00739	Haptoglobin-related protein	HPR	0.17372	0.0403
Q9NPH3	Interleukin-1 receptor accessory protein	IL1RAP	0.15913	0.0362
P19827	Inter-alpha-trypsin inhibitor heavy chain H1	ITIH1	0.136853	0.0452
P09871	Complement C1s subcomponent	C1S	0.128955	0.0357
P02774	Vitamin D-binding protein	GC	−0.17533	0.00304
Q16610	Extracellular matrix protein 1	ECM1	−0.19871	0.0156
P49908	Selenoprotein P	SELENOP	−0.20905	0.0066
P13591	Neural cell adhesion molecule 1	NCAM1	−0.21476	0.0342
P61769	Beta-2-microglobulin	B2M	−0.23769	0.0255
P01871	Immunoglobulin heavy constant mu	IGHM	−0.24231	0.00603
P61626	Lysozyme C	LYZ	−0.24325	0.0183
P06396	Gelsolin	GSN	−0.26045	0.0189
Q03591	Complement factor H-related protein 1	CFHR1	−0.26836	0.0426
P06727	Apolipoprotein A-IV	APOA4	−0.29485	0.0106
Q96QR1	Secretoglobin family 3A member 1	SCGB3A1	−0.31331	0.0292
P01619	Immunoglobulin kappa variable 3–20	IGKV3-20	−0.33987	0.00893
O14786	Neuropilin-1	NRP1	−0.3454	0.0153
Q6UXB8	Peptidase inhibitor 16	PI16	−0.42234	0.0214
Q9NPY3	Complement component C1q receptor	CD93	−0.43177	0.0065
P09211	Glutathione S-transferase P	GSTP1	−0.43399	0.0435
P08519	Apolipoprotein(a)	LPA	−0.44072	0.0234
Q12805	EGF-containing fibulin-like extracellular matrix protein 1	EFEMP1	−0.44838	0.00641
P49747	Cartilage oligomeric matrix protein	COMP	−0.48649	0.00115
P47756	F-actin-capping protein subunit beta	CAPZB	−0.55793	0.0442
P12814	Alpha-actinin-1	ACTN1	−0.57426	0.0327
P21291	Cysteine and glycine-rich protein 1	CSRP1	−0.63131	0.0104
P24844	Myosin regulatory light polypeptide 9	MYL9	−0.63826	0.04
Q15113	Procollagen C-endopeptidase enhancer 1	PCOLCE	−1.15362	8.69 × 10⁻^9^

**Table 7:** Changes in plasma protein expression during acute phase compared to stabilization phase in TS patients. A comprehensive list of proteins characterized by their accession number, protein name, and symbol. The variation in expression levels is quantified using the Log2FC (Log2 fold change), with positive values indicating upregulation in the acute phase compared to the stabilization phase, and negative values indicating downregulation. The statistical significance of these changes is represented by the *T*-test *p*-values.

Accession No.	Protein Name	Protein Symbol	Log2FC	*p*-Value
P0DJI9	Serum amyloid A-2 protein	SAA2	3.586989	0.0133
P02741	C-reactive protein	CRP	2.040108	0.000303
P0DJI8	Serum amyloid A-1 protein	SAA1	1.869815	0.0423
Q13418	Integrin-linked protein kinase	ILK	0.885547	0.0319
Q08830	Fibrinogen-like protein 1	FGL1	0.786728	0.00509
P55774	C-C motif chemokine 18	CCL18	0.782166	0.018
P18428	Lipopolysaccharide-binding protein	LBP	0.649046	0.011
O14950;P19105	Myosin regulatory light chain 12B	MYL12B	0.527264	0.0464
P01033	Metalloproteinase inhibitor 1	TIMP1	0.473518	0.0251
P05556	Integrin beta-1	ITGB1	0.447755	0.0455
O95810	Caveolae-associated protein 2	CAVIN2	0.305359	0.0396
P01019	Angiotensinogen	AGT	0.286575	0.00233
P33908	Mannosyl-oligosaccharide 1,2-alpha-mannosidase IA	MAN1A1	0.236024	0.0128
P05160	Coagulation factor XIII B chain	F13B	−0.15496	0.0435
P02745	Complement C1q subcomponent subunit A	C1QA	−0.21297	0.00893
P00748	Coagulation factor XII	F12	−0.22025	0.0456
P01700	Immunoglobulin lambda variable 1–47	IGLV1-47	−0.22973	0.0347
P19823	Inter-alpha-trypsin inhibitor heavy chain H2	ITIH2	−0.23373	0.0315
P02786	Transferrin receptor protein 1	TFRC	−0.26734	0.0246
P04196	Histidine-rich glycoprotein	HRG	−0.28371	0.0129
P49747	Cartilage oligomeric matrix protein	COMP	−0.33966	0.0454
P06727	Apolipoprotein A-IV	APOA4	−0.5037	0.0205
Q13740	CD166 antigen	ALCAM	−0.53791	0.0127
Q15113	Procollagen C-endopeptidase enhancer 1	PCOLCE	−0.551	0.00924
P23083	Immunoglobulin heavy variable 1–2	IGHV1-2	−0.55475	0.0115

## Data Availability

The mass spectrometry and Spectronaut analysis files have been deposited with the ProteomeXchange consortium via the MassIVE repository (dataset identifier MSV000094514) and are accessible on PRIDE via ID PXD051367 [53].

## Supplementary Materials

The following supporting information can be downloaded at https://www.mdpi.com/article/10.3390/cells13211764/s1, Table S1. The table summarizes the plasma proteins differentially regulated between patients with STEMI and patients with TS in the acute phase. Table S2. This table outlines differentially regulated plasma proteins between patients with STEMI and patients with TS in the stabilization phase. Table S3. The table presents differentially regulated plasma proteins by comparing patients with STEMI from the acute to stabilization phases. Table S4. This table outlines plasma protein changes between acute and stabilization phases in TS patients.

## Abbreviations

MI	Myocardial infarction
TS	Takotsubo syndrome
PCI	Percutaneous coronary intervention
STEMI	ST-elevation myocardial infarction
RI	Reperfusion injury
EDTA	Ethylenediaminetetraacetic acid
DTT	DL-dithiothreitol
IAA	Iodoacetamide
LC-MS/MS	Tandem mass spectrometry
GO	Gene Ontology
FDR	False discovery rate
KEGG	Kyoto Encyclopedia of Genes and Genomes
HDL	High-density lipoprotein
COPD	Chronic obstructive pulmonary disease
hs-cTnI	High-sensitivity cardiac troponin I
hs-cTnT	High-sensitivity cardiac troponin T
NT-proBNP	N-terminal pro B-type natriuretic peptide
IQR	Interquartile range
HDL	High-density lipoprotein
LDL	Low-density lipoprotein
HF	Heart failure
SMD	Standardized mean difference
BMI	Body mass index
ACEi	Angiotensin-converting enzyme inhibitor
ARB	Angiotensin receptor blocker
ASA	Acetylsalicylic acid
OAC	Oral anticoagulant
P2Y12i	P2Y12 receptor inhibitor
TIA	Transient ischemic attack
