# Supplementary material for: Comparative Analysis of Plasma Protein Dynamics in Women with ST-Elevation Myocardial Infarction and Takotsubo Syndrome
Source: Cells. 2024 Oct 24;13(21):1764. doi: 10.3390/cells13211764 (PMC11545104; doi:10.3390/cells13211764)
Supplement: Supplementary file 1 [file cells-13-01764-s001.zip › cells-3212883-supplementary.pdf]

SUPPLEMENTARY DATA

Table S1. The table summarizes the plasma proteins differentially regulated between patients with STEMI and patients with TS in the acute phase. It illustrates enriched pathways/functions, associated proteins, fold enrichment, and statistical significance (FDR) for GO Biological Process, GO Cellular Component, GO Molecular Function, and KEGG pathways. Upregulated/downregulated proteins are presented separately, offering insights into molecular changes between conditions.

**Upregulated proteins in STEMI acute vs. TS acute in GO Biological Process**					
Enrichment FDR	nProteins	Pathway Proteins	Fold Enrichment	Pathway	Proteins
1.69E-06	3	24	241.225	GO:0006957 complement activation alternative pathway	C8B C8A C8G
6.20E-08	4	33	233.9152	GO:0019835 cytolysis	C8B C6 C8A C8G
2.38E-07	4	54	142.9481	GO:0006958 complement activation classical pathway	C8B C6 C8A C8G
0.043376	1	16	120.6125	GO:0006825 copper ion transport	CP
3.84E-07	4	67	115.2119	GO:0002455 humoral immune response mediated by circulating immunoglobulin	C8B C6 C8A C8G
7.22E-07	4	84	91.89524	GO:0006956 complement activation	C6 C8A C8B C8G
5.24E-06	4	152	50.78421	GO:0016064 immunoglobulin mediated immune response	C8B C6 C8A C8G
5.80E-06	4	162	47.64938	GO:0019724 B cell mediated immunity	C8B C6 C8A C8G
**Upregulated proteins in STEMI acute vs. TS acute in GO Cellular Component**					
Enrichment FDR	nProteins	Pathway Proteins	Fold Enrichment	Pathway	Proteins
3.58E-09	4	26	261.7385	GO:0046930 pore complex	C8B C6 C8A C8G
0.000128	3	141	36.19787	GO:0072562 blood microparticle	CP C8A C8G
0.047179	1	67	25.39254	GO:1904724 tertiary granule lumen	CAMP
0.047179	1	82	20.74756	GO:0035580 specific granule lumen	CAMP
**Upregulated proteins in STEMI acute vs. TS acute in GO Molecular Function**					
Enrichment FDR	nProteins	Pathway Proteins	Fold Enrichment	Pathway	Proteins
0.002086	2	31	127.9642	GO:0001848 complement binding	C8G C8A
0.039983	1	17	116.6732	GO:0016722 oxidoreductase activity acting on metal ions	CP
0.039983	1	18	110.1914	GO:0019841 retinol binding	C8G
0.039983	1	18	110.1914	GO:0052745 inositol phosphate phosphatase activity	MINPP1

0.039983	1	23	86.23671	GO:0030021 extracellular matrix structural constituent conferring compression re	PRG4
0.039983	1	31	63.98208	GO:0004181 metallocarboxypeptidase activity	CPN1
0.039983	1	34	58.3366	GO:0030247 polysaccharide binding	PRG4
0.039983	1	40	49.58611	GO:0001530 lipopolysaccharide binding	CAMP
0.039983	1	40	49.58611	GO:0005501 retinoid binding	C8G
0.039983	1	40	49.58611	GO:0019840 isoprenoid binding	C8G
**Upregulated proteins in STEMI acute vs. TS acute in KEGG Pathways**					
Enrichment FDR	nProteins	Pathway Proteins	Fold Enrichment	Pathway	Proteins
8.85E-06	4	85	48.10588	Path:hsa04610 Complement and coagulation cascades	C6 C8A C8B C8G
2.83E-05	4	135	30.28889	Path:hsa05322 Systemic lupus erythematosus	C6 C8A C8B C8G
0.000403	3	102	30.06618	Path:hsa05146 Amoebiasis	C8A C8B C8G
**Downregulated proteins in STEMI acute vs. TS acute in GO Biological Process**					
Enrichment FDR	nProteins	Pathway Proteins	Fold Enrichment	Pathway	Proteins
0.007207	2	47	164.2383	GO:0033032 reg. of myeloid cell apoptotic proc.	ADIPOQ ITPKB
0.007207	2	54	142.9481	GO:0033028 myeloid cell apoptotic proc.	ADIPOQ ITPKB
0.007207	2	61	126.5443	GO:1904705 reg. of vascular associated smooth muscle cell proliferation	ADIPOQ MMP2
0.007207	2	69	111.8725	GO:0014823 response to activity	MMP2 ADIPOQ
0.01238	2	99	77.97172	GO:0045638 negative reg. of myeloid cell differentiation	ADIPOQ ITPKB
0.036774	1	57	67.71228	GO:0001580 detection of chemical stimulus involved in sensory perception of bitt	PIGR
0.036774	1	58	66.54483	GO:0001541 ovarian follicle development	MMP2
0.024193	2	160	48.245	GO:0048659 smooth muscle cell proliferation	ADIPOQ MMP2
**Downregulated proteins in STEMI acute vs. TS acute in GO Molecular Function**					
Enrichment FDR	nProteins	Pathway Proteins	Fold Enrichment	Pathway	Proteins
0.029491	1	16	185.9479	GO:0016840 carbon-nitrogen lyase activity	APMAP

0.029491	1	20	148.7583	GO:0019763 immunoglobulin receptor activity	PIGR
0.029491	1	22	135.2348	GO:0033691 sialic acid binding	ADIPOQ
0.032889	1	33	90.15657	GO:0001968 fibronectin binding	MMP2
0.032889	1	41	72.56504	GO:0016776 phosphotransferase activity phosphate group as acceptor	ITPKB
0.036032	1	54	55.09568	GO:0098632 cell-cell adhesion mediator activity	ROBO4
0.037119	1	65	45.77179	GO:0098631 cell adhesion mediator activity	ROBO4

Table S2. This table outlines differentially regulated plasma proteins between patients with STEMI and patients with TS in the stabilization phase. It details enriched pathways/functions, proteins involved, fold enrichment, and statistical significance (FDR) for GO Biological Process, GO Cellular component, GO Molecular Function, and KEGG pathways. The data is categorized into upregulated and downregulated proteins, providing insights into comparative molecular changes.

**Upregulated proteins in STEMI stabilization vs. TS stabilization in GO Biological Process**					
Enrichment FDR	nProteins	Pathway Proteins	Fold Enrichment	Pathway	Proteins
0.02457	1	20	321.6333	GO:0010310 reg. of hydrogen peroxide metabolic proc.	HP
0.00077	2	57	225.7076	GO:0006953 acute-phase response	SAA1 HP
0.002018	2	130	98.9641	GO:0002526 acute inflammatory response	HP SAA1
0.02457	1	69	93.22705	GO:0031638 zymogen activation	HP
0.02457	1	106	60.68553	GO:0032732 positive reg. of interleukin-1 production	SAA1
0.02457	1	109	59.01529	GO:0032963 collagen metabolic proc.	PCOLCE
0.02457	1	123	52.2981	GO:0030593 neutrophil chemotaxis	SAA1
0.02457	1	143	44.98368	GO:0030168 platelet activation	SAA1
0.02457	1	154	41.77056	GO:0032612 interleukin-1 production	SAA1
**Upregulated proteins in STEMI stabilization vs. TS stabilization in GO Cellular Component**					
Enrichment FDR	nProteins	Pathway Proteins	Fold Enrichment	Pathway	Proteins
5.15E-05	2	24	472.5833	GO:0071682 endocytic vesicle lumen	SAA1 HP
0.014878	1	35	162.0286	GO:0034364 high-density lipoprotein particle	SAA1
0.014878	1	44	128.8864	GO:0034358 plasma lipoprotein particle	SAA1
0.014878	1	44	128.8864	GO:1990777 lipoprotein particle	SAA1
0.014878	1	47	120.6596	GO:0032994 protein-lipid complex	SAA1

0.01619	1	67	84.64179	GO:1904724 tertiary granule lumen	HP
0.01619	1	80	70.8875	GO:0005881 cytoplasmic microtubule	SAA1
0.01619	1	82	69.15854	GO:0035580 specific granule lumen	HP
0.024659	1	141	40.21986	GO:0072562 blood microparticle	HP
**Upregulated proteins in STEMI stabilization vs. TS stabilization in GO Molecular Function**					
Enrichment FDR	nProteins	Pathway Proteins	Fold Enrichment	Pathway	Proteins
0.019278	1	46	129.3551	GO:0016504 peptidase activator activity	PCOLCE
0.02006	1	75	79.33778	GO:0005518 collagen binding	PCOLCE
0.001661	2	189	62.96649	GO:0008201 heparin binding	PCOLCE SAA1
0.02006	1	96	61.98264	GO:0016209 antioxidant activity	HP
0.0311	1	187	31.81996	GO:0005201 extracellular matrix structural constituent	PCOLCE
**Downregulated proteins in STEMI stabilization vs. TS stabilization in GO Biological Process**					
Enrichment FDR	nProteins	Pathway Proteins	Fold Enrichment	Pathway	Proteins
2.15E-07	4	37	231.8078	GO:0034368 protein-lipid complex remodeling	PLTP NA APOB AGT
2.15E-07	4	37	231.8078	GO:0034369 plasma lipoprotein particle remodeling	PLTP NA APOB AGT
2.15E-07	4	39	219.9202	GO:0034367 protein-containing complex remodeling	PLTP NA APOB AGT
2.09E-05	3	32	201.0208	GO:0010743 reg. of macrophage derived foam cell differentiation	APOB AGT ADIPOQ
2.84E-05	3	38	169.2807	GO:0090077 foam cell differentiation	APOB AGT ADIPOQ
6.12E-07	4	53	161.8281	GO:0071827 plasma lipoprotein particle organization	PLTP NA APOB AGT
6.39E-07	4	56	153.1587	GO:0071825 protein-lipid complex subunit organization	PLTP NA APOB AGT
6.09E-08	5	79	135.7103	GO:0097006 reg. of plasma lipoprotein particle levels	PLTP NA APOB ADIPOQ AGT
9.46E-07	4	64	134.0139	GO:0033344 cholesterol efflux	NA PLTP ADIPOQ APOB
**Downregulated proteins in STEMI stabilization vs. TS stabilization in GO Cellular Component**					
Enrichment FDR	nProteins	Pathway Proteins	Fold Enrichment	Pathway	Proteins
0.000254	2	19	179.0842	GO:0034362 low-density lipoprotein particle	APOB NA
1.40E-05	3	35	145.8257	GO:0034364 high-density lipoprotein particle	NA PLTP APOB
0.000325	2	24	141.775	GO:0071682 endocytic vesicle lumen	APOB NA
0.000325	2	27	126.0222	GO:0034361 very-low-density lipoprotein particle	APOB NA
0.000325	2	27	126.0222	GO:0034385 triglyceride-rich plasma lipoprotein particle	APOB NA
1.40E-05	3	44	115.9977	GO:0034358 plasma lipoprotein particle	APOB NA PLTP

1.40E-05	3	44	115.9977	GO:1990777 lipoprotein particle	APOB NA PLTP
1.40E-05	3	47	108.5936	GO:0032994 protein-lipid complex	APOB NA PLTP
0.02388	1	17	100.0765	GO:0042101 T cell receptor complex	ALCAM
0.000517	2	36	94.51667	GO:0005790 smooth endoplasmic reticulum	NA APOB
**Downregulated proteins in STEMI stabilization vs. TS stabilization in GO Molecular Function**					
Enrichment FDR	nProteins	Pathway Proteins	Fold Enrichment	Pathway	Proteins
0.001397	2	23	155.2261	GO:0120020 cholesterol transfer activity	APOB PLTP
0.001397	2	24	148.7583	GO:0120015 sterol transfer activity	APOB PLTP
0.027747	1	18	99.17222	GO:0008525 phosphatidylcholine transporter activity	PLTP
0.001815	2	37	96.49189	GO:0015248 sterol transporter activity	APOB PLTP
0.00266	2	50	71.404	GO:0120013 lipid transfer activity	APOB PLTP
0.027747	1	31	57.58387	GO:0016645 oxidoreductase activity acting on the CH-NH group of donors	BLVRB
0.027747	1	32	55.78438	GO:0016628 oxidoreductase activity acting on the CH-CH group of donors NAD or NA	BLVRB
0.009904	2	106	33.68113	GO:0004867 serine-type endopeptidase inhibitor activity	TFPI AGT
0.001397	3	176	30.42784	GO:0005319 lipid transporter activity	APOB PLTP NA
0.0127	2	130	27.46308	GO:0005179 hormone activity	AGT ADIPOQ
**Downregulated proteins in STEMI stabilization vs. TS stabilization in KEGG Pathways**					
Enrichment FDR	nProteins	Pathway Proteins	Fold Enrichment	Pathway	Proteins
0.02606	2	51	40.08824	Path:hsa04979 Cholesterol metabolism	APOB PLTP
0.028028	2	75	27.26	Path:hsa03320 PPAR signaling pathway	PLTP ADIPOQ

Table S3. The table presents differentially regulated plasma proteins by comparing patients with STEMI from the acute to stabilization phases. It highlights enriched pathways/functions, proteins, fold enrichment, and statistical significance (FDR) for GO Biological Process, GO Cellular Component, GO Molecular Function, and KEGG pathways. The information is divided into upregulated and downregulated proteins, offering insights into molecular changes across the two time points.

**Upregulated proteins in STEMI acute vs. STEMI stabilization in GO Biological Process**					
Enrichment FDR	nProteins	Pathway Proteins	Fold Enrichment	Pathway	Proteins
1.25E-07	6	37	65.19595	GO:0034368 protein-lipid complex remodeling	CETP APOA5 NA APOE APOB AGT

1.25E-07	6	37	65.19595	GO:0034369 plasma lipoprotein particle remodeling	CETP APOA5 NA APOE APOB AGT
1.25E-07	6	39	61.85256	GO:0034367 protein-containing complex remodeling	CETP APOA5 NA APOE APOB AGT
4.82E-07	6	53	45.51415	GO:0071827 plasma lipoprotein particle organization	CETP APOA5 NA APOB APOE AGT
4.82E-07	6	54	44.6713	GO:0006958 complement activation classical pathway	CRP MASP2 C8B MBL2 C8G C1S
5.17E-07	6	56	43.07589	GO:0071825 protein-lipid complex subunit organization	CETP APOA5 NA APOB APOE AGT
1.03E-06	6	64	37.69141	GO:0033344 cholesterol efflux	APOA5 NA PON1 APOE APOB CETP
1.21E-06	6	67	36.00373	GO:0002455 humoral immune response mediated by circulating immunoglobulin	CRP MASP2 C8B MBL2 C8G C1S
2.61E-07	7	84	33.50347	GO:0006956 complement activation	MASP2 CRP FCN3 MBL2 C8B C8G C1S
2.24E-06	7	130	21.6484	GO:0002526 acute inflammatory response	LBP HPR VNN1 CRP SAA2 SAA1 MBL2
**Upregulated proteins in STEMI acute vs. STEMI stabilization in GO Cellular Component**					
Enrichment FDR	nProteins	Pathway Proteins	Fold Enrichment	Pathway	Proteins
2.20E-17	10	35	101.2679	GO:0034364 high-density lipoprotein particle	CETP APOA5 NA PON1 APOL1 APOE HPR APOB SAA2 SAA1
4.04E-18	11	44	88.60938	GO:0034358 plasma lipoprotein particle	APOB CETP APOA5 APOE NA PON1 APOL1 PCYOX1 HPR SAA2 SAA1
4.04E-18	11	44	88.60938	GO:1990777 lipoprotein particle	APOB CETP APOA5 APOE NA PON1 APOL1 PCYOX1 HPR SAA2 SAA1
6.08E-18	11	47	82.95346	GO:0032994 protein-lipid complex	APOB CETP APOA5 APOE NA PON1 APOL1 PCYOX1 HPR SAA2 SAA1
6.34E-10	6	27	78.76389	GO:0034361 very-low-density lipoprotein particle	APOB NA APOL1 APOA5 PCYOX1 APOE

6.34E-10	6	27	78.76389	GO:0034385 triglyceride-rich plasma lipoprotein particle	APOB APOE NA APOL1 APOA5 PCYOX1
3.06E-06	4	24	59.07292	GO:0071682 endocytic vesicle lumen	APOB APOE SAA1 NA
9.45E-05	3	19	55.96382	GO:0034362 low-density lipoprotein particle	APOB NA APOE
1.28E-12	11	141	27.65115	GO:0072562 blood microparticle	PON1 ITIH1 APOL1 BCHE APOE AGT FCN3 C8G CPN2 C1S HPR
0.001755	4	124	11.43347	GO:0005796 Golgi lumen	F7 F9 PROC PROZ
**Upregulated proteins in STEMI acute vs. STEMI stabilization in GO Molecular Function**					
Enrichment FDR	nProteins	Pathway Proteins	Fold Enrichment	Pathway	Proteins
3.79E-05	4	24	63.30142	GO:0050750 low-density lipoprotein particle receptor binding	APOB APOE APOA5 CRP
0.000733	3	23	49.54024	GO:0120020 cholesterol transfer activity	APOB CETP APOE
0.000733	3	24	47.47606	GO:0120015 sterol transfer activity	APOB CETP APOE
0.007918	2	17	44.68335	GO:0001846 opsonin binding	CRP MASP2
0.001285	3	31	36.75566	GO:0001848 complement binding	CRP C8G MASP2
0.001696	3	37	30.79528	GO:0015248 sterol transporter activity	APOB CETP APOE
0.003006	3	50	22.78851	GO:0120013 lipid transfer activity	APOB CETP APOE
0.001893	4	106	14.3324	GO:0004867 serine-type endopeptidase inhibitor activity	TFPI AGT NA ITIH1
0.001696	5	189	10.04784	GO:0008201 heparin binding	APOB APOA5 APOE SAA1 NA
**Upregulated proteins in STEMI acute vs. STEMI stabilization in GO KEGG Pathways**					
Enrichment FDR	nProteins	Pathway Proteins	Fold Enrichment	Pathway	Proteins
4.43E-12	10	85	34.36134	Path:hsa04610 Complement and coagulation cascades	MASP2 F5 F7 F9 MBL2 PROC TFPI C1S C8B C8G
0.015425	3	51	17.18067	Path:hsa04979 Cholesterol metabolism	CETP APOB APOE
**Downregulated proteins in STEMI acute vs. STEMI stabilization in GO Biological Process**					
Enrichment FDR	nProteins	Pathway Proteins	Fold Enrichment	Pathway	Proteins
0.035937	2	22	79.7438	GO:1900120 reg. of receptor binding	NA NRP1
0.035937	2	28	62.65584	GO:0003416 endochondral bone growth	COMP ECM1
0.035937	2	30	58.47879	GO:0051016 barbed-end actin filament capping	CAPZB GSN

0.035937	2	31	56.59238	GO:0098868 bone growth	COMP ECM1
0.03894	2	34	51.59893	GO:0002063 chondrocyte development	COMP ECM1
0.027495	3	79	33.3107	GO:0070527 platelet aggregation	MYL9 CSRP1 COMP
0.035937	3	104	25.30332	GO:0034109 homotypic cell-cell adhesion	MYL9 CSRP1 COMP
0.001215	5	192	22.84328	GO:0055001 muscle cell development	ACTN1 MYL9 CSRP1 COMP PI16
0.035937	3	121	21.74831	GO:0002062 chondrocyte differentiation	EFEMP1 COMP ECM1
0.035937	3	143	18.40242	GO:0030168 platelet activation	MYL9 CSRP1 COMP
**Downregulated proteins in STEMI acute vs. STEMI stabilization in GO Cellular Component**					
Enrichment FDR	nProteins	Pathway Proteins	Fold Enrichment	Pathway	Proteins
0.009857	2	21	73.64935	GO:0042827 platelet dense granule	ECM1 SELENOP
0.013535	2	44	35.15083	GO:0034358 plasma lipoprotein particle	APOA4 LPA
0.013535	2	44	35.15083	GO:1990777 lipoprotein particle	APOA4 LPA
0.013535	2	47	32.90716	GO:0032994 protein-lipid complex	APOA4 LPA
0.001914	4	141	21.9381	GO:0072562 blood microparticle	APOA4 GC GSN CFHR1
0.023119	2	74	20.90049	GO:0001725 stress fiber	ACTN1 MYL9
0.009857	3	118	19.66063	GO:0030863 cortical cytoskeleton	ACTN1 GSN CAPZB
0.023119	2	82	18.86142	GO:0032432 actin filament bundle	ACTN1 MYL9
0.012424	3	141	16.45358	GO:0030018 Z disc	ACTN1 CSRP1 MYL9
0.013075	3	155	14.96745	GO:0031674 I band	ACTN1 CSRP1 MYL9
**Downregulated proteins in STEMI acute vs. STEMI stabilization in GO Molecular Function**					
Enrichment FDR	nProteins	Pathway Proteins	Fold Enrichment	Pathway	Proteins
0.03098	2	46	35.27866	GO:0008307 structural constituent of muscle	CSRP1 MYL9
0.04171	2	70	23.18312	GO:0004714 transmembrane receptor protein tyrosine kinase activity	EFEMP1 NRP1
0.04171	2	75	21.63758	GO:0005518 collagen binding	PCOLCE COMP
0.04171	2	76	21.35287	GO:0017022 myosin binding	MYL9 GSN
0.002454	4	187	17.35634	GO:0005201 extracellular matrix structural constituent	COMP PCOLCE EFEMP1 ECM1
0.002454	4	189	17.17268	GO:0008201 heparin binding	COMP PCOLCE NRP1 LPA
0.049313	2	96	16.90436	GO:0016209 antioxidant activity	GSTP1 APOA4

Table S4. This table outlines plasma protein changes between acute and stabilization phases in TS patients. It includes enriched pathways/functions, individual proteins, fold enrichment, and statistical significance for GO and KEGG pathways, categorized into upregulated and downregulated proteins.

**Upregulated proteins in TS acute vs. TS stabilization in GO Biological Process**					
Enrichment FDR	nProteins	Pathway Proteins	Fold Enrichment	Pathway	Proteins
0.005388	2	18	164.9402	GO:0010958 reg. of amino acid import across plasma membrane	AGT ITGB1
0.005388	2	20	148.4462	GO:0032930 positive reg. of superoxide anion generation	CRP AGT
0.005388	2	21	141.3773	GO:0008228 opsonization	LBP CRP
0.005388	2	25	118.7569	GO:0051957 positive reg. of amino acid transport	AGT ITGB1
2.49E-05	4	57	104.1727	GO:0006953 acute-phase response	LBP CRP SAA2 SAA1
0.000348	4	130	45.67574	GO:0002526 acute inflammatory response	LBP CRP SAA2 SAA1
0.005388	3	123	36.20638	GO:0030593 neutrophil chemotaxis	NA LBP SAA1
0.005388	3	130	34.2568	GO:0007229 integrin-mediated signaling pathway	ITGB1 ILK TIMP1
0.005388	3	148	30.09044	GO:0071621 granulocyte chemotaxis	NA LBP SAA1
**Upregulated proteins in TS acute vs. TS stabilization in GO Cellular Component**					
Enrichment FDR	nProteins	Pathway Proteins	Fold Enrichment	Pathway	Proteins
0.006429	2	35	74.78242	GO:0034364 high-density lipoprotein particle	SAA2 SAA1
0.006429	2	44	59.48601	GO:0034358 plasma lipoprotein particle	SAA2 SAA1
0.006429	2	44	59.48601	GO:1990777 lipoprotein particle	SAA2 SAA1
0.006429	2	47	55.68903	GO:0032994 protein-lipid complex	SAA2 SAA1
0.009728	2	74	35.37006	GO:0001725 stress fiber	MYL12B ILK
0.009728	2	74	35.37006	GO:0097517 contractile actin filament bundle	MYL12B ILK
0.009728	2	82	31.91932	GO:0032432 actin filament bundle	MYL12B ILK
0.009728	2	82	31.91932	GO:0042641 actomyosin	MYL12B ILK
**Upregulated proteins in TS acute vs. TS stabilization in KEGG pathways**					
Enrichment FDR	nProteins	Pathway Proteins	Fold Enrichment	Pathway	Proteins

0.035556	2	77	26.55195	Path:hsa05100 Bacterial invasion of epithelial cells	ILK ITGB1
0.035556	2	90	22.71667	Path:hsa05410 Hypertrophic cardiomyopathy	AGT ITGB1
0.035556	2	96	21.29688	Path:hsa05414 Dilated cardiomyopathy	AGT ITGB1
0.0391	2	114	17.93421	Path:hsa04670 Leukocyte transendothelial migration	MYL12B ITGB1
0.021458	3	181	16.94337	Path:hsa04360 Axon guidance	MYL12B ILK ITGB1
0.0391	2	124	16.4879	Path:hsa04611 Platelet activation	MYL12B ITGB1
**Downregulated proteins in TS acute vs. TS stabilization in GO Biological Process**					
Enrichment FDR	nProteins	Pathway Proteins	Fold Enrichment	Pathway	Proteins
0.002924	2	17	227.0353	GO:0072378 blood coagulation fibrin clot formation	F12 F13B
0.002924	2	19	203.1368	GO:0051917 reg. of fibrinolysis	HRG F12
0.002924	2	20	192.98	GO:0072376 protein activation cascade	F12 F13B
0.003463	2	28	137.8429	GO:0042730 fibrinolysis	HRG F12
0.003463	2	29	133.0897	GO:0030194 positive reg. of blood coagulation	HRG F12
0.003463	2	29	133.0897	GO:1900048 positive reg. of hemostasis	HRG F12
0.000699	3	52	111.3346	GO:1900047 negative reg. of hemostasis	HRG F12 COMP
0.000978	3	73	79.30685	GO:1900046 reg. of hemostasis	HRG F12 COMP
0.003463	3	176	32.89432	GO:0007568 aging	TFRC COMP C1QA
**Downregulated proteins in TS acute vs. TS stabilization in GO Cellular Component**					
Enrichment FDR	nProteins	Pathway Proteins	Fold Enrichment	Pathway	Proteins
**1.92E-05**	**4**	141	48.26383	GO:0072562 blood microparticle	TFRC APOA4 HRG ITIH2
**Downregulated proteins in TS acute vs. TS stabilization in GO Molecular Function**					
Enrichment FDR	nProteins	Pathway Proteins	Fold Enrichment	Pathway	Proteins
0.001462	2	19	208.7836	GO:0043395 heparan sulfate proteoglycan binding	COMP HRG
0.001889	2	37	107.2132	GO:0043394 proteoglycan binding	COMP HRG
0.005844	2	75	52.89185	GO:0005518 collagen binding	PCOLCE COMP

0.0093	2	106	37.42348	GO:0004867 serine-type endopeptidase inhibitor activity	HRG ITIH2
0.001779	3	189	31.48325	GO:0008201 heparin binding	HRG COMP PCOLCE
0.023712	2	187	21.21331	GO:0005201 extracellular matrix structural constituent	COMP PCOLCE
**Downregulated proteins in TS acute vs. TS stabilization in KEGG Pathways**					
Enrichment FDR	nProteins	Pathway Proteins	Fold Enrichment	Pathway	Proteins
0.000589	3	85	41.23361	Path:hsa04610 Complement and coagulation cascades	F12 F13B C1QA
